# Association analysis and functional follow-up identified common variants of *JAG1* accounting for risk to biliary atresia

**DOI:** 10.3389/fgene.2023.1186882

**Published:** 2023-05-15

**Authors:** Mei-Rong Bai, Hao-Yue Pei, Ying Zhou, Huan-Lei Song, Wei-Hua Pan, Yi-Ming Gong, Wen-Jie Wu, Wen-Wen Yu, Meng-Meng Cui, Bei-Lin Gu, Xun Chu, Wei Cai

**Affiliations:** ^1^ Department of Pediatric Surgery, Xinhua Hospital Affiliated to Shanghai Jiaotong University School of Medicine, Shanghai, China; ^2^ Shanghai Institute of Pediatric Research, Shanghai, China; ^3^ Shanghai Key Laboratory of Pediatric Gastroenterology and Nutrition, Shanghai, China

**Keywords:** biliary atresia, *JAG1*, single nucleotide polymorphisms, zebrafish, bile ducts

## Abstract

**Background:** Biliary atresia (BA) is a destructive, obliterative cholangiopathy characterized by progressive fibro-inflammatory disorder and obliteration of intra- and extrahepatic bile ducts. The *Jagged1* (*JAG1*) gene mutations have been found in some isolated BA cases. We aim to explore the association of common variants in *JAG1* with isolated BA risk in the Chinese Han population.

**Methods:** We genotyped 31 tag single nucleotide polymorphisms covering the *JAG1* gene region in 333 BA patients and 1,665 healthy controls from the Chinese population, and performed case-control association analysis. The expression patterns of *JAG1* homologs were investigated in zebrafish embryos, and the roles of *jag1*a and *jag1b* in biliary development were examined by morpholino knockdown in zebrafish.

**Results:** Single nucleotide polymorphisms rs6077861 [*P*_*Allelic*_ = 1.74 × 10^−4^, odds ratio = 1.78, 95% confidence interval: 1.31–2.40] and rs3748478 (*P*_*Allelic*_ = 5.77 × 10^−4^, odds ratio = 1.39, 95% confidence interval: 1.15–1.67) located in the intron region of *JAG1* showed significant associations with BA susceptibility. The *JAG1* homologs, *jag1a* and *jag1b* genes were expressed in the developing hepatobiliary duct of zebrafish, especially at 72 and 96 h postfertilization. Knockdown of both *jag1a* and *jag1b* led to poor biliary secretion, sparse intrahepatic bile duct network and smaller or no gallbladders compared with control embryos in the zebrafish model.

**Conclusion:** Common genetic variants of *JAG1* were associated with BA susceptibility. Knockdown of *JAG1* homologs led to defective intrahepatic and extrahepatic bile ducts in zebrafish. These results suggest that *JAG1* might be implicated in the etiology of BA.

## 1 Introduction

Biliary atresia (BA) is an inflammatory cholangiopathy of infancy characterized by progressive occlusion and fibrosclerosis of intra- and extrahepatic bile ducts (Hartley et al., 2009). The incidence of BA has significant geographic variation ranging from 1:5,000–1:10,000 in Asia (Wada et al., 2007; Chiu et al., 2013) to an estimated 1:15,000 in the United States and 1:19,000 in the Netherlands (Asai et al., 2015). BA occurs seasonally, with the highest incidence rate between December and March and has a female predominance of approximately 1.25 times that of males in syndromic BA (Asai et al., 2015). The definitive diagnosis of BA is usually made by surgery, liver biopsy and surgical cholangiography including collapsed gallbladder and invisible common bile duct or common hepatic duct (Li et al., 2008). The Kasai hepatic portoenterostomy is the standard therapy for BA to restore bile flow. However, even with a successful Kasai hepatic portoenterostomy, most infants have persisting liver dysfunction and more than 70% patients eventually develop cirrhosis and still need liver transplantation in the later stage (Sokol et al., 2007). Some BA patients are accompanied by a range of abnormalities including asplenia or polysplenia, vascular abnormalities, situs inversus and cardiac anomalies. Depending on the absence or presence of accompanied abnormalities, BA is usually classified as syndromic (about 15% of cases) and isolated (approximately 85% of cases) types (Lakshminarayanan and Davenport, 2016).

The pathogenesis of isolated BA is poorly understood, which is proposed to be caused by genetic proposition and environmental factors such as a virus and toxin (Asai et al., 2015; Bezerra et al., 2018). The most widely accepted hypothesis of initial hepatobiliary injury involves potential viral infections in the early perinatal period for non-syndromic or acquired BA (Malik et al., 2020). The viral infections and immune response might impair the integrity of the primary ciliary structure and the function on the top surface of bile duct cells, which was considered an important pathogenic factor for BA (Chu et al., 2012; Malik et al., 2020; Lam et al., 2021). Recent genome-wide association studies (GWASs) identified common variants in *ADD3*, *GPC1*, and *ARF6* associated with isolated BA (Garcia-Barceló et al., 2010; Leyva-Vega et al., 2010; Cheng et al., 2013; Cui et al., 2013; Ningappa et al., 2015). Mutations in *PKD1L1* and *MAN1A2* have been linked to syndromic BA (Berauer et al., 2019; So et al., 2020). Mutations in *EFEMP1* and three ciliary genes, including *PCNT*, *KIF3B*, and *TTC17* have been found in both syndromic and non-syndromic BA patients (Chen et al., 2018; Lam et al., 2021). The lack of each of these genes impeded bile flow and intrahepatic bile duct development in zebrafish, which further emphasized the importance of variants in these gene in BA pathogenesis (Malik et al., 2020). Mutations in the *Jagged1* (*JAG1*) gene have been found in non-syndromic BA (Kohsaka et al., 2002; Cheng et al., 2017; Sangkhathat et al., 2018). Kohsaka et al. (2002) explored the *JAG1* gene mutations in 102 cases of the extrahepatic biliary atresia (EHBA), and found that the most *JAG1* gene mutations were all missense and sporadic and were generally detected in severely ill patients who have received liver transplantation under 5 years old. However, none of the 9 EHBA patients with the *JAG1* mutations showed any of the 5 major symptoms of Alagille syndrome (ALGS), nor any pathological findings similar to ALGS after 3 years of follow-up, which suggested the *JAG1* gene abnormalities may be an aggravating factor in EHBA. Interestingly, approximately 95% of ALGS patients had mutations in *JAG1* (Oda et al., 1997).

ALGS is an autosomal-dominant multisystem disorder defined clinically by at least three of the following five major features: Chronic cholestasis or intrahepatic bile duct paucity, congenital cardiac disease, skeletal anomalies (typically butterfly vertebrae), ocular abnormalities and characteristic facial feature (Mitchell et al., 2018). It was reported that about 89% of the ALGS patients had cholestasis and about 75% had bile duct paucity (Mitchell et al., 2018). Given that cholestasis and bile duct abnormality are commonly seen in both ALGS and BA (Verkade et al., 2016; Feldman and Sokol, 2019), some ALGS patients display a considerable overlap with BA.

The *JAG1* gene influenced duct formation during the embryonal stage (Louis et al., 1999). The Notch signaling pathway regulated by JAG1 was critical for postnatal bile duct growth and bile duct branching morphology (Loomes et al., 2007; Roos et al., 2022). Early lack of bile ducts led to cholestasis, which subsequently stimulated the proliferation of bile ducts (Tharehalli et al., 2018). Considering *JAG1* mutations found in BA patients and its critical roles in the development of liver and bile duct, we investigated whether common variants of *JAG1* were associated with BA risk through a case-control association study and examined the role of *jag1* in biliary tract development in zebrafish model.

## 2 Materials and methods

### 2.1 Subjects

We recruited 333 BA patients from 2008 to 2018 who were diagnosed with BA by intraoperative cholangiography and pathology of liver tissue after ultrasonography evaluation in Xinhua hospital affiliated to Shanghai Jiao Tong University School of Medicine as described in our previous study (Bai et al., 2020). All the patients were isolated BA cases without the presence of other malformations. The clinical data of BA patients, including age at surgery, gender and liver biochemical indicators were collected, which were provided in our previous study (Bai et al., 2020). Healthy individuals who visited Xinhua hospital for routine health check were recruited as controls. A group of 1,665 unrelated healthy individuals without BA, other congenital diseases, autoimmune, or liver disease were enrolled as described previously (Bai et al., 2020). All the case and control participants in the study were biologically unrelated individuals. Written informed consent for the collection of blood samples was obtained from participants or their legal guardians. Peripheral blood samples were collected in standard EDTA tubes. Genomic DNA was extracted using QIAamp DNA Blood Mini Kit according to the manufacturer’s protocol (Qiagen, Hilden, Germany). The study was conducted in accordance with the Declaration of Helsinki (version 2002) and was approved by the Institutional Review Board of Xinhua Hospital affiliated to Shanghai Jiaotong University School of Medicine (IRB: XHEC-H-2022-001-1).

### 2.2 SNP selection, genotyping, and functional annotation

Tag single nucleotide polymorphisms (SNPs) for *JAG1* were selected using the SNPinfo Web Server with minor allele frequency ≥0.01 and *r* ^2^ ≥ 0.8 as the selection criteria based on the HapMap Chinese Han in Beijing data. A total of 31 tag SNPs were selected (Supplementary Table S1) and genotyped in 333 cases and 1,665 controls using Fluidigm 96.96 Dynamic Array IFCs (Fluidigm, San Francisco, CA, United States) (Supplementary Table S2). The HaploRegv4.1 database was used to predict the function of the associated SNPs (Supplementary Table S3).

### 2.3 Real time polymerase chain reaction (RT-PCR) and quantitative RT-PCR (q-PCR) experiments in zebrafish

All zebrafish experiments were carried out on the AB line zebrafish. All procedures were conducted in full accordance with the institutional guidelines approved by the Animal care and Use Committee of Xinhua Hospital (XHEC-WSJSW-2018-029). Total RNA was extracted from pools of 25 zebrafish embryos at different developmental time points using TRIzol reagent (Invitrogen, Cat # 9109, United States). The Primescript RT Master Kit (Takara, Catalyst # RR036A, Japan) was used to synthesize cDNA. To quantify the relative expression of *jag1a* and *jag1b*, q-PCR was performed using SYBR Green Master Mix (Applied Biosystems, Catalyst # A25742, United States) with fluorescent tags located on QuantStudio Dx Real Time PCR Instrument (Applied Biosystems, CA, United States). The primers for q-PCR were listed in Supplementary Table S4. The 18s ribosomal RNA (*18-s*) gene was used for standardization (McCurley and Callard, 2008). The relative expression levels of each sample with three independent triplicates were calculated using the RQ formula (RQ = 2^−ΔΔCT^). To demonstrate the effectiveness of the splice-blocking morpholinos (SBMOs), RT-PCR was performed using Premix Taq™ (Takara, Cat#RR902A, Japan). The primers for RT-PCR were listed in Supplementary Table S5.

### 2.4 Riboprobe synthesis and whole-mount *in situ* hybridization (WISH)

We performed PCR to obtain the specific fragments of *jag1a* and *jag1b*, cloned the PCR fragments into pGEM-T Easy Vector (Promega, WI, United States), and generated digoxigenin-labeled (Roche Applied Science, Penzberg, Germany) RNA probes by *in vitro* transcription. PCR primers used to generate riboprobes were shown in Supplementary Table S6. WISH was performed as previously described (Lu et al., 2021). We used *jag1a* and *jag1b* antisense RNA probes at the concentration of 3 ng/μl to hybridize with zebrafish embryos.

### 2.5 Morpholino (MO) microinjection

MOs were designed to act at the translational start site or splicing acceptor site of the *jag1a* or *jag1b* and ordered from Gene Tools, LLC (Philomath, OR, United States). Standard MO was used as negative control. MOs dissolved in distilled water were microinjected into the yolk of 1–4 cell stage embryos as previously described (Cui et al., 2013; Lu et al., 2021). The sequences of MOs were listed in Supplementary Table S7. We performed dose-dependent experiments on *jag1a*-MO and *jag1b*-MO, and found the dose with the best effect was 2 ng.

### 2.6 N-((6-(2,4-dinitrophenyl) amino) hexanoyl)-1-palmitoyl-2-BODIPY-FL-pentanoyl-sn-glycero-3-phosphoethanolamine (PED-6) treatment

The feeding of PED-6 (Invitrogen, Cat#D23739, United States), a fluorescent phosphor-lipase A2 reporter, was performed on 5 days postfertilization (5dpf) embryos at a concentration of 0.1 mg/mL for no less than 2 hours (Cui et al., 2013). We determined the metabolism of fluorescent lipids or biliary secretion by observing the size of the gallbladder and the concentration of PED-6 in the gallbladder using a stereo fluorescence microscope (Nikon SMZ25, Japan). Each group of zebrafish embryos was scored as “normal”, “faint” and “no” based on the size of the gallbladder and the concentration of PED-6 therein (Cui et al., 2013).

### 2.7 Whole-mount immunofluorescence

After fixation in methanol/dimethyl sulfoxide, whole-mount cytokeratin immunostaining was performed on 5dpf larvae as previously described (Cui et al., 2013). Larvae were incubated with primary antibody against Keratin K18 (PROGEN, Cat#61028, Germany) at a dilution of 1:100 and Alexa Fluor 488 AffiniPure goat anti-mouse IgG (H + L) (YEASEN, Cat#33206ES60, China) diluted at 1:500 was used as secondary antibody. Immuno-stained larvae were examined using a laser scanning confocal microscope (Leica TCS SP8, Germany), and images were processed using Adobe Photoshop. ImageJ software was used to quantify the gallbladder area.

### 2.8 Statistical analysis

Hardy-Weinberg equilibrium tests for the genotype distribution of each SNP in the case and control groups was performed and case-control association analysis was conducted using PLINK 1.09. A study-wide level *p*-value <0.0016 (0.05/31) was considered statistically significant. All statistical analysis was performed by GraphPad Prism 8 software unless specified. The quantitative data was presented as the mean ± standard error of the mean (SEM). When the data conformed to the homogeneity of the variance, the two tailed Student’s *t*-test was used, otherwise the Mann-Whitney test was used. The uptake of PED-6 in larvae was analyzed by chi-square test. All *p*-values were bilateral, *p* < 0.05 was considered statistically significant.

## 3 Results

### 3.1 Association of *JAG1* SNPs with BA

All 31 tag SNPs conformed to Hardy-Weinsberg equilibrium and the minor allele frequencies were all above 0.01 in 1,665 controls. The allelic and genotypic distribution between cases and controls are shown in Supplementary Tables S1, S2, respectively. After multiple testing, we found that two SNPs reached the study-level significance, and the allele and genotype distribution of these two SNPs are shown in Table 1.

**TABLE 1:** Two tag SNPs showed association with sporadic BA risk in 333 patients and 1,665 controls.

SNP	Genotype/Allele	Number (frequency)		Cases vs*.* controls	
		Cases	Controls	*P*	Or 95% CI
rs6077861	A	615 (0.92)	2901 (0.87)	1.74 × 10^−4^	1.78 (1.31–2.40)
	T	51 (0.08)	427 (0.13)		
	A/A	286 (0.86)	1263 (0.76)	3.22 × 10^−4^	
	A/T	43 (0.13)	375 (0.23)		
	T/T	4 (0.01)	26 (0.02)		
rs3748478	T	190 (0.29)	746 (0.22)	5.77 × 10^−4^	1.39 (1.15–1.67)
	C	474 (0.71)	2582 (0.78)		
	T/T	19 (0.06)	73 (0.04)	9.65 × 10^−4^	
	T/C	152 (0.46)	600 (0.36)		
	C/C	161 (0.48)	991 (0.60)		

SNP, single nucleotide polymorphism; OR, odds ratio; CI, confidence interval.

Two tag SNPs located in the intron region of *JAG1* showed significant association with BA susceptibility. Rs6077861 was the most associated SNP. The major allele A of rs6077861 was associated with BA risk [*P*_Allelic_ = 1.74 × 10^−4^, odds ratio (OR) = 1.78, 95% confidence interval (95% CI): 1.31–2.40]. The frequency of rs6077861 allele A was 0.923 in cases and 0.8717 in controls (Supplementary Table S1). The second association signal was rs3748478. The minor allele T of rs3748478 was associated with BA risk (*P*_Allelic_ = 5.77 × 10^−4^, OR = 1.39, 95% CI: 1.15–1.67). The frequency of rs3748478 allele T was 0.286 in cases and 0.224 in controls (Supplementary Table S1). The genotype distributions of the two SNPs in BA patients were also significantly different from controls (*P*_Genotypic-rs6077861_ = 3.22 × 10^−4^; *P*_Genotypic-rs3748478_ = 9.65 × 10^−4^; Supplementary Table S2). The two associated SNPs were in extreme low linkage disequilibrium (*r* ^2^ < 0.01), which might represent an independent association signal.

### 3.2 Functional annotation

Both rs6077861 and rs3748478 fell into strong enhancer activity regions and DNase I hypersensitivity sites and altered the sequences of regulatory motifs (Supplementary Table S3). Rs6077861 was an expression quantitative trait locus of *JAG1* in the whole blood (*p* = 1.50 × 10^−4^) (Supplementary Table S3).

### 3.3 Spatiotemporal expression of *jag1a* and *jag1b* in developing zebrafish embryos

**FIGURE 1:**
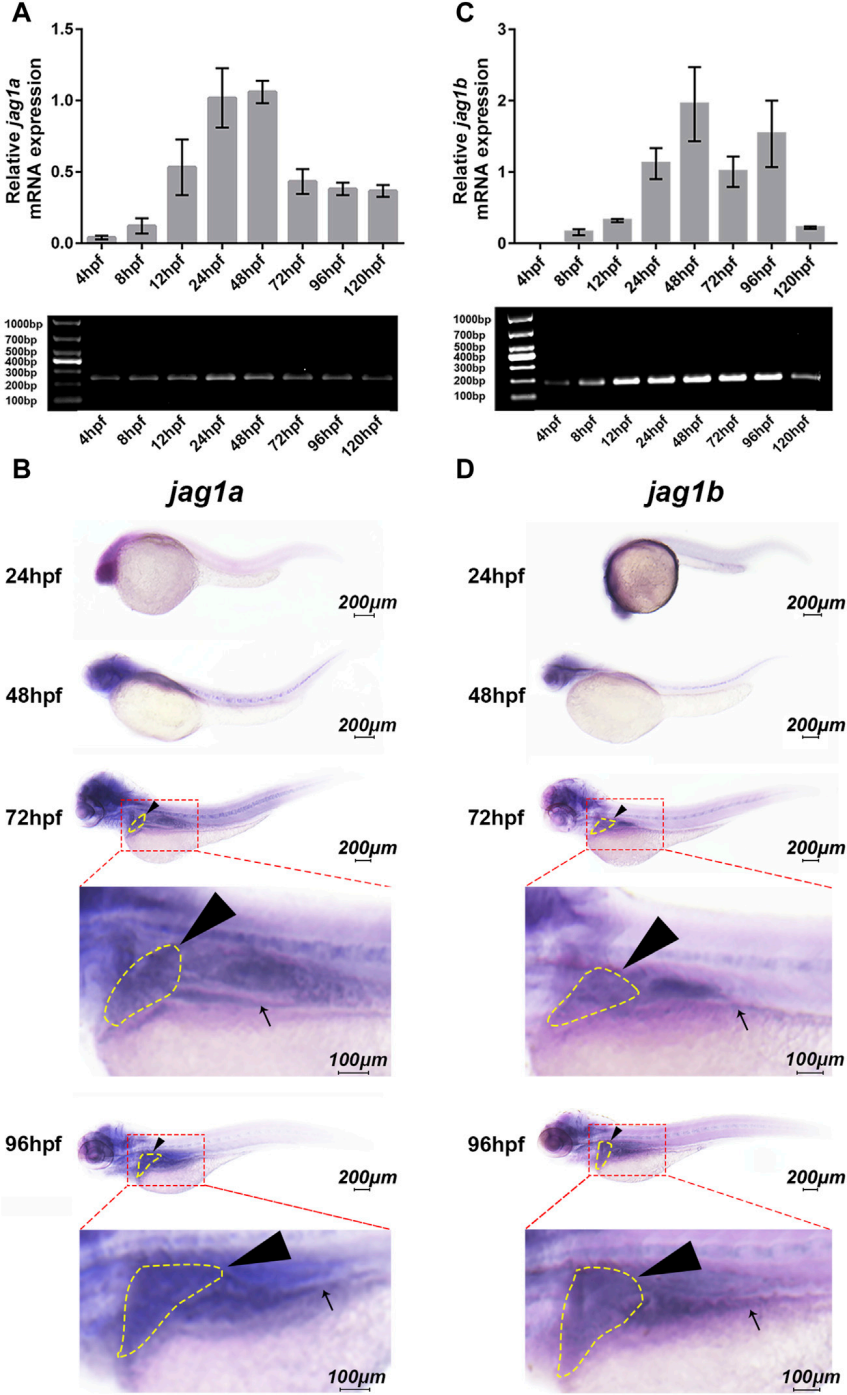
Spatiotemporal expression patterns of *jag1a* and *jag1b* in zebrafish larvae. **(A, C)** The relative transcript levels of *jag1a* and *jag1b* were analyzed by q-PCR during embryogenesis from 4hpf to 120hpf. **(B, D)** The spatial expression patterns of *jag1a* and *jag1b* were detected by WISH in 24hpf, 48hpf, 72hpf, and 96hpf zebrafish embryos. Black arrowheads and arrows mark liver and intestine respectively. Scale bar = 200 μm and 100 μm. All the data are shown as the mean ± SEM. q-PCR, quantitative polymerase chain reaction; hpf, hour postfertilization; WISH, whole-mount *in situ* hybridization; SEM, standard error of the mean.

We first explored whether *jag1a* and *jag1b* were expressed in the developing hepatobiliary duct of zebrafish. The expression of *jag1a* was present at 12 h postfertilization (hpf), peaked at 24-48hpf, then decreased and maintained a stable expression level (Figure 1A). Similarly, the *jag1b* mRNA showed obvious expression at 12hpf, maintained high expression at 24-96hpf, and decreased expression at 5dpf (Figure 1C).

To further explore the spatial expression patterns of *jag1a* and *jag1b* in the developing liver of zebrafish, WISH analysis was performed using antisense riboprobes. The results showed that *jag1a* was heavily expressed in the head at 24–96hpf, and moderate expression was observed in the liver and intestine, especially at 72hpf and 96hpf (Figure 1B). The expression pattern of *jag1b* was similar to that of *jag1a*, which was heavily expressed in the liver and intestine of zebrafish at 72hpf and 96hpf (Figure 1D), an important period in which bile duct growth is active, accompanied by rapid proliferation of cholangiocytes and formation of nascent ducts (Matthews et al., 2009). Therefore, we knocked down the expression of *jag1a* and *jag1b* to evaluate the influence on the biliary development.

### 3.4 The expressions of *jag1a* and *jag1b* were effectively knocked down with MOs

**FIGURE 2:**
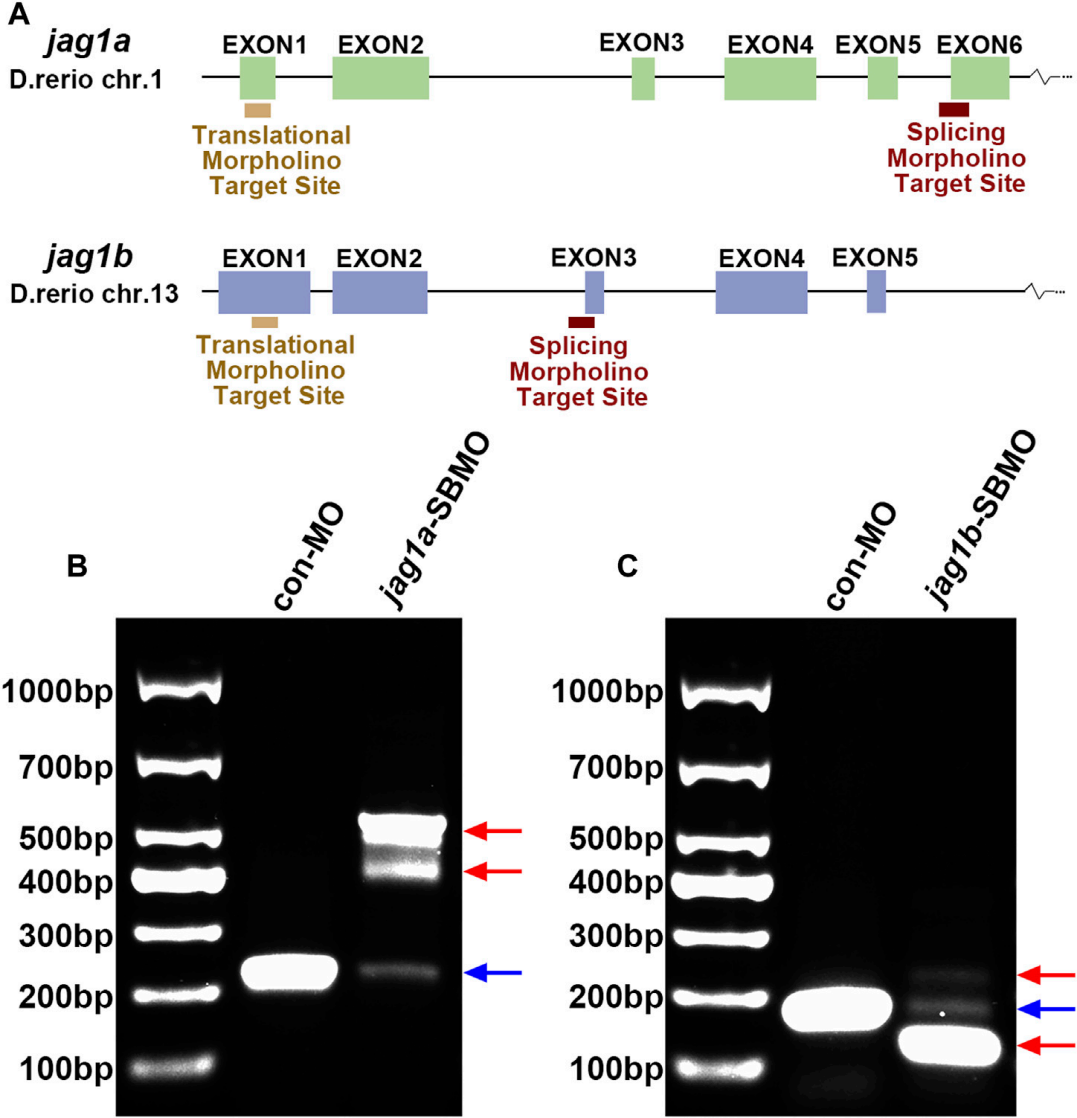
The expression of *jag1a* or *jag1b* was effectively knocked down with MO. **(A)** The target sites of *jag1a*-SBMO and *jag1b*-SBMO are at I5E6 and I2E3, respectively. The target sites of *jag1a*-TBMO and *jag1b*-TBMO are both located in E1. **(B, C)** RT-PCR confirmed the effectiveness of *jag1a*-SBMO and *jag1b*-SBMO, which demonstrated by the appearance of abnormally spliced fragments marked by red arrows. Abbreviations: SBMO, splice-blocking morpholino; TBMO, translation-blocking morpholino; E, exon; I, intron; RT-PCR, reverse transcription-polymerase chain reaction.

We used two non-overlapping MOs for each gene: A translation-blocking MO (TBMO) and a SBMO. The SBMOs were complementary to the exon 6 splice acceptor site (intron5exon6, I5E6) of *jag1a* and the acceptor site of exon3 (intron2exon3, I2E3) of *jag1b*, respectively. Both TBMOs acted on the first exons (Figure 2A). To identify the effectiveness of SBMO, we performed RT-PCR and gel electrophoresis, which showed that injection of *jag1a* and *jag1b* SBMOs induced decreased expression of *jag1* and alteration of transcription, as demonstrated by the appearance of abnormally spliced fragments marked by red arrows (Figures 2B, C).

**FIGURE 3:**
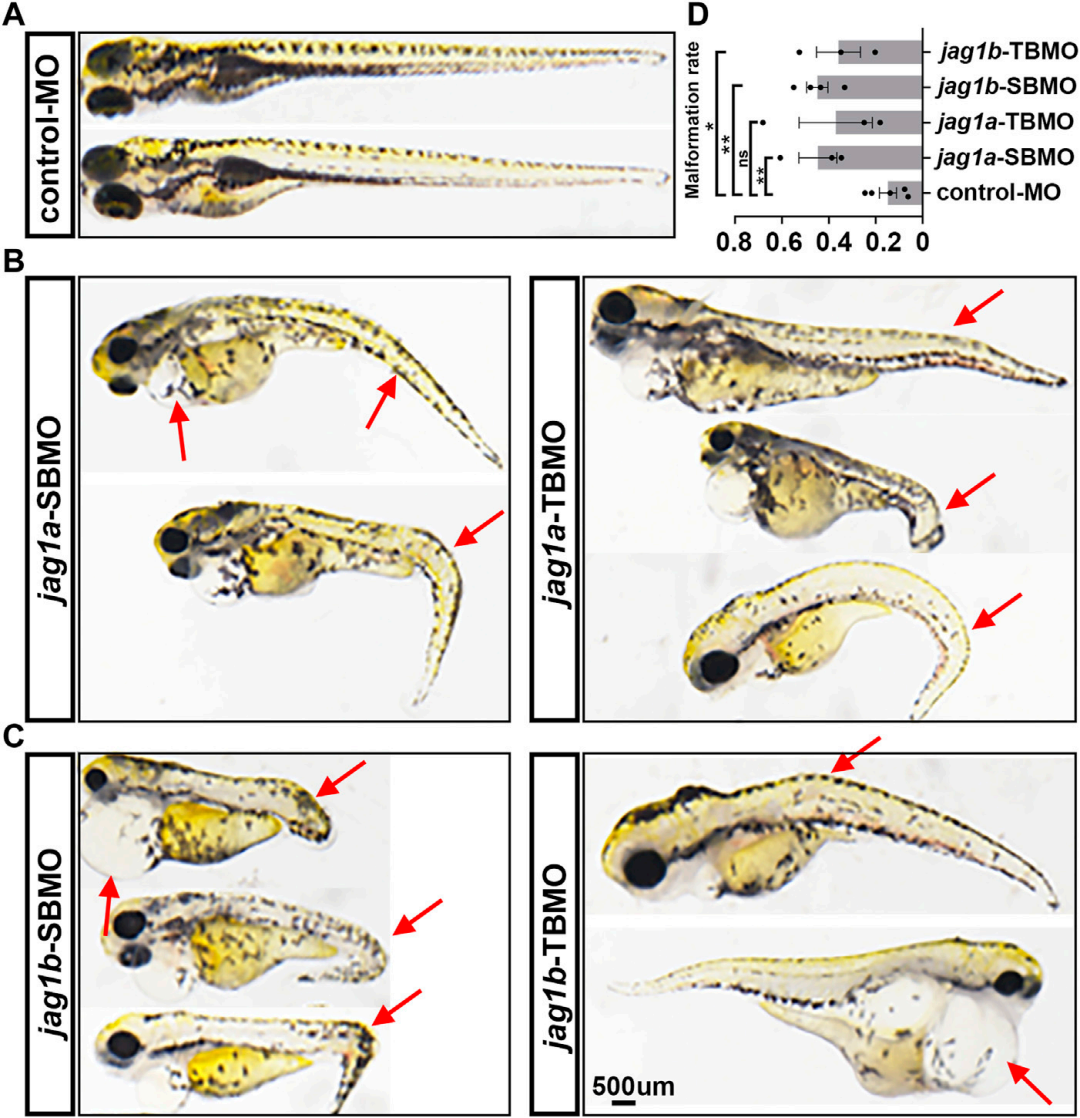
Morphological abnormality of 5dpf zebrafish injected with *jag1a* and *jag1b* MOs. **(A)** Presentative morphology of zebrafish injected with control MO; **(B)** The typical deformed zebrafish injected with *jag1a* MOs; **(C)** The typical deformed zebrafish injected with *jag1b* MOs; **(D)** The statistics of malformation rate of *jag1a* and *jag1b* morphants were higher than that of control MO group. Except for *jag1a*-TBMO group, there was significant difference between them and control MO group. Scale bar = 500 μm. All the data are shown as the mean ± SEM. *p < 0.05; **p < 0.01; ***p < 0.001; ****p < 0.0001. dpf, day postfertilization; MO, morpholino; SEM, standard error of the mean.

The 5dpf embryos injected with *jag1a*-MOs and *jag1b*-MOs both exhibited significant morphological abnormalities, including pericardial edema, spinal and tail curvature and body shortening (Figures 3B, C), compared to embryos injected with control MO (Figure 3A). The statistics of malformation rate of *jag1a* and *jag1b* morphants were higher than that of control MO group. Except for *jag1a*-TBMO group, there was significant difference between them and control MO group (Figure 3D).

### 3.5 Knockdown of *jag1a* or *jag1b* led to biliary defects and dysfunction

**FIGURE 4:**
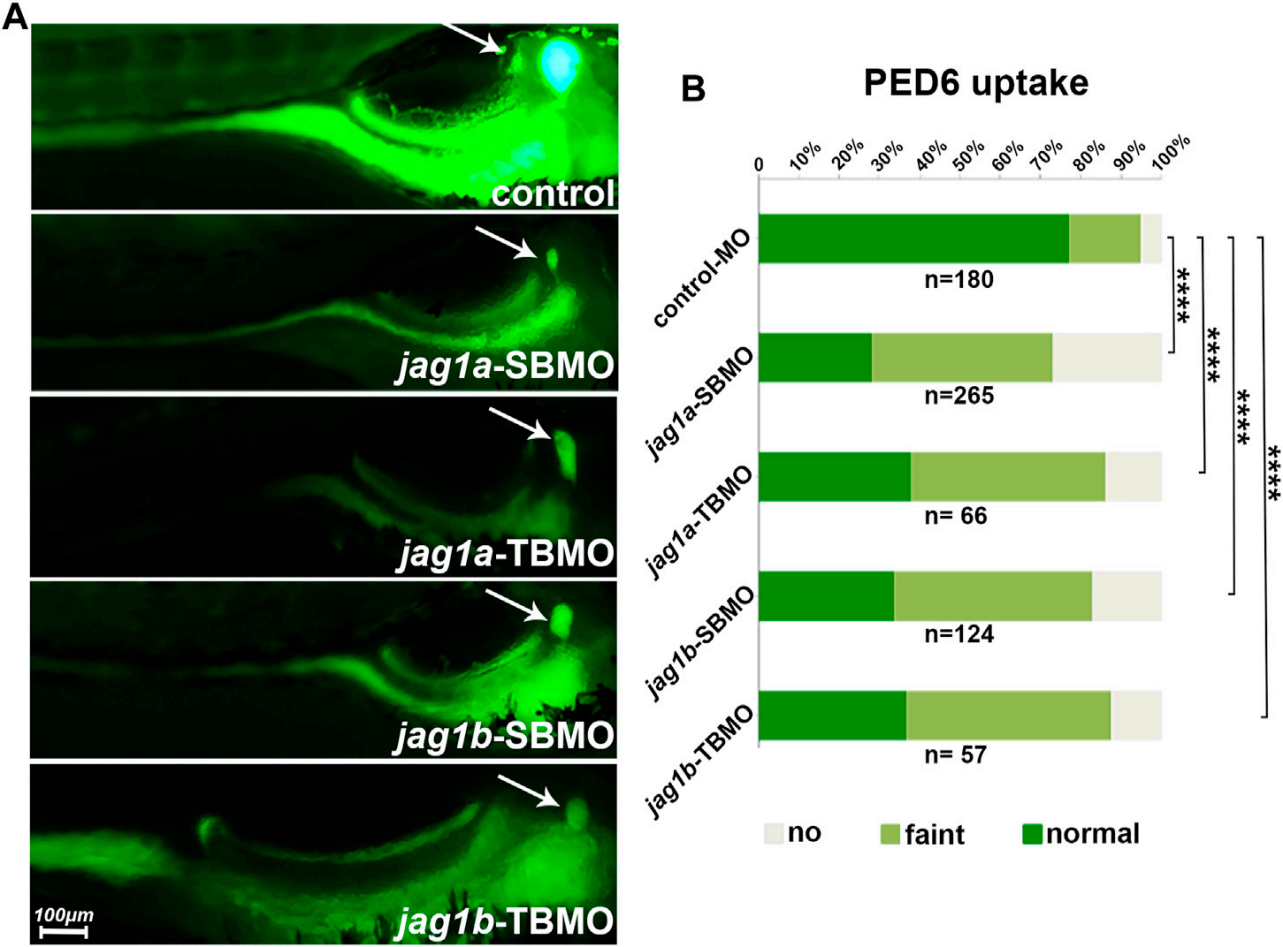
Knockdown of *jag1a* or *jag1b* led to biliary dysfunction in zebrafish. **(A)** The right lying view of 5dpf zebrafish larvae after swallowing PED-6 showed that the gallbladders of *jag1a* or *jag1b* morphants were weaker and smaller than those of embryos injected with control MO. **(B)** Quantitative analysis showed that the accumulation of PED-6 in the gallbladders of *jag1a* or *jag1b* morphants were significantly reduced (*p* < 0.0001, χ^2^ test). Scale bar = 100 μm *p < 0.05; **p < 0.01; ***p < 0.001; ****p < 0.0001. Abbreviations: dpf, day post fertilization; PED-6, N-((6-(2,4-dinitrophenyl) amino) hexanoyl)-1-palmitoyl-2-BODIPY-FL-pentanoyl-sn-glycero-3-phosphoethanolamine.

PED-6 is absorbed in the intestine after swallowing and enriches in the gallbladder after being processed by the liver and secreted with bile (Cui et al., 2013). The accumulation of PED-6 in the gallbladder is reduced if the intrahepatic bile ducts are abnormal (Cui et al., 2013). We conducted PED-6 swallowing to determine the effect of *jag1* on bile secretion function. We found that *jag1a* and *jag1b* morphants showed poor bile drainage compared to embryos injected with control MO (Figure 4A) judged by gallbladder size and PED-6 enrichment within the gallbladder. There were statistically significant differences between the two morphants and the control (*p* < 0.0001, Figure 4B).

**FIGURE 5:**
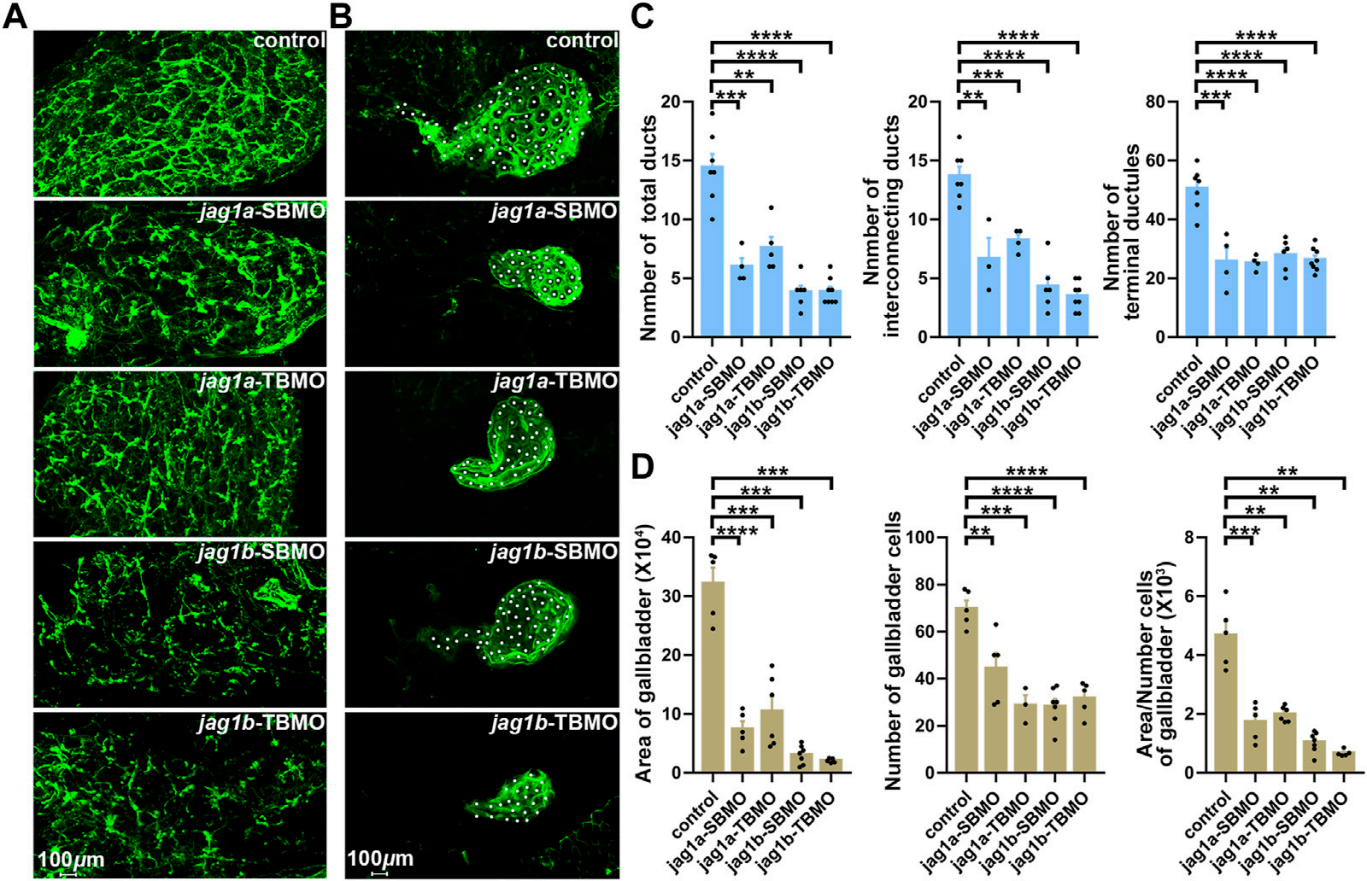
Knockdown of *jag1a* or *jag1b* led to biliary defects in zebrafish. **(A)** Confocal projection on 5dpf control larvae and *jag1* morphants stained with cytokeratin 18 showed that the intrahepatic bile ducts of *jag1a* or *jag1b* morphants were sparse compared to controls. **(B)** Confocal projection on 5dpf control larvae and *jag1* morphants stained with cytokeratin 18 showed that gallbladders of *jag1a* or *jag1b* morphants were smaller or even no compared to those of embryos injected with control MO. Gallbladder cells were noted by white dots. **(C)** Quantitative analysis of the numbers of total ducts, interconnecting ducts and terminal ductules showed that intrahepatic bile ducts were significantly reduced in both *jag1a* and *jag1b* morphants compared with the embryos injected with control MO. **(D)** Quantitative analysis of the number of gallbladder cells, the measured area of gallbladders and the mean area of individual gallbladder cell showed that both *jag1a* and *jag1b* morphants had smaller gallbladders and individual gallbladder cells compared to the embryos injected with control MO. Scale bar = 100 μm. All the data are shown as the mean ± SEM. *p < 0.05; **p < 0.01; ***p < 0.001; ****p < 0.0001. dpf, day post fertilization; SEM, standard error of the mean.

We further examined the intra- and extrahepatic bile duct anatomy by cytokeratin immunostaining and found that both *jag1a* and *jag1b* morphants had sparse intrahepatic bile ducts and smaller or even no gallbladders compared to controls (Figures 5A, B). We counted the numbers of total ducts numbers, interconnecting ducts and terminal ductules, and found these ducts were significantly reduced in both *jag1a* and *jag1b* morphants compared with the embryos injected with control MO (*p* < 0.01, Figure 5C). In addition, we counted the number of gallbladder cells, and measured the area of gallbladder and the mean area of individual gallbladder cell. Compared to the embryos injected with control MO, both *jag1a* and *jag1b* morphants had smaller gallbladders and individual gallbladder cell (*p* < 0.01, Figure 5D). These results suggested that insufficiencies of *jag1* led to developmental anomalies of intrahepatic and extrahepatic biliary, supporting a potential role of *jag1* in BA etiology.

## 4 Discussion

In this study, we performed an association analysis for common variation of *JAG1* with BA susceptibility and found that two variants (rs6077861 and rs3748478) were associated with BA in the Chinese population. Knockdown of the expression of homologues of *JAG1* in zebrafish led to biliary defects and small or even absent gallbladder. These results supported a role of *JAG1* in the development of BA.

*JAG1* rare mutations were repeatedly found in BA patients. Kohsaka et al. (2002) detected *JAG1* missense mutations in 9 of 102 cases of EHBA, and *JAG1* mutations were generally found in critically ill patients under 5 years of age who received liver transplantation. Moreover, none of the 9 EHBA patients with *JAG1* mutations showed any of the five main symptoms of ALGS or any identical pathological features after 3 years of follow-up. These patients could be diagnosed as EHBA rather than atypical ALGS. Cheng et al. (2017) performed a cross-sectional observational study and discovered one *de novo* microdeletion of *JAG1* in a BA patient diagnosed as type III BA by intraoperative cholangiography. Surasak *et al.* investigated rare nonsynonymous variants of 19 genes associated with infantile cholestasis using whole exome sequencing in 20 surgically and histopathologically diagnosed BA cases, and found *JAG1* was the most frequent mutated gene (Sangkhathat et al., 2018). Our current study found that common SNPs in *JAG1* gene region were associated with risk to sporadic BA, which reinforced the role of *JAG1* in the etiology of BA development. Of note, for the number of cases was small, further independent replication was needed.

The occurrence of BA has regional and racial differences (Asai et al., 2015; Bezerra et al., 2018). The frequencies of the risk allele A for rs6077861 and the risk allele T for rs3748478 in the Asian population are higher than that of other populations, which might have a role in higher prevalence of BA in the Asian population compared to other populations. Both rs6077861 and rs3748478 are located in the enhancer activity regions of *JAG1* gene. The enhancer is a key gene regulatory element that controls the spatiotemporal gene expression program specific to cell type and its function is the basis of the regulatory process of establishing gene expression patterns in cells (Ong and Corces, 2011; Schoenfelder and Fraser, 2019). It usually enhances gene transcription by combining with specific transcription factors, and enhances the expression of target genes through long-range chromosome interaction (Schoenfelder and Fraser, 2019). The sequence variation and genome rearrangement of enhancer may be the basis of disease susceptibility and developmental abnormalities, though altering the transcription level of target genes (Schoenfelder and Fraser, 2019). Therefore, it could be speculated that rs6077861 and rs3748478 may lead to a decreased expression of *JAG1*, underlying the susceptibility of BA.

Zebrafish emerged as a useful model in studying the pathogenesis of BA. PED-6, a fluorescent lipid reporter, is swallowed, absorbed by the intestine, processed through the liver, and excreted into bile, concentrating in the gallbladder. It is often used for examination of biliary function of zebrafish, and decreased gallbladder uptake of PED-6 would be caused by the maldevelopment of gallbladder (Cheng et al., 2013). BODIPY C5 (4,4-Difluoro-5,7-Dimethyl-4-Bora-3a,4a-Diaza-s-Indacene-3-Pentanoic Acid) is another fluorescent lipid reporter, which is often used to investigate bile canaliculi and intrahepatic bile conduits of zebrafish in addition to the gallbladder (So et al., 2018). The roles of susceptibility genes from recent GWASs in the pathogenesis of BA were evaluated in zebrafish model. In 2010, Leyva Vega et al. identified *GPC1* as a BA susceptibility gene for deletions in 2q37.3 containing *GPC1* in BA patients (Leyva-Vega et al., 2010). Knockdown of *gpc1* in zebrafish led to developmental biliary defects and activation of the Hedgehog pathway (Cui et al., 2013). *ADD3* was identified in a GWAS in the Chinese population, and knockdown of *add3* in zebrafish resulted biliary trees defects (Garcia-Barceló et al., 2010; Cheng et al., 2013; Tang et al., 2016). In 2015, Ningappa et al. conducted genotyping in the American population and found a significant association between *ARF6* and BA (Ningappa et al., 2015). Knockdown of *arf6* in zebrafish resulted in sparse intrahepatic bile duct network, defects in bile duct epithelial cells and poor bile excretion, suggesting that *ARF6* might be a susceptibility gene for BA (Ningappa et al., 2015). In 2020, a GWAS study for BA requiring liver transplantation identified the susceptibility gene *MAN1A2*. Knockdown of *man1a2* in zebrafish impaired the development of biliary network (So et al., 2020). In addition, *man1a2* morphant zebrafish displayed ciliary dysgenesis in Kupffer’s vesicle, and cardiac and liver heterotaxy, indicating a novel molecular mechanism for multiple system defects in BA (So et al., 2020).

Knockdown of *jag1* in zebrafish caused the poor formation of intrahepatic bile duct network and the dysfunction of bile excretion, manifested by the lack of PED-6 accumulation in the gallbladder. In addition, *jag1* knockdown also impaired the development of extrahepatic bile ducts including the reduction of area, cell numbers and unicellular area of gallbladder. The *jag1* morphants mimic syndromes found in some BA patients including failure to excrete radionuclides into extrahepatic bile duct during hepatobiliary scanning, failed surgical drainage, extrahepatic bile duct occlusion and small or absent gallbladders, and lack of bile duct (Li et al., 2008). The intrahepatic bile ducts could not be examined before the onset of jaundice or before the occurrence of extrahepatic biliary tract obstruction in BA patients. Therefore, it was now unclear whether the alterations in the intrahepatic bile ducts of BA children were primary lesions or caused by the obstruction of extrahepatic bile duct. Continuous biopsies from four children diagnosed with EHBA showed that the intrahepatic bile duct deficiency without fibrosis preceded bile duct hyperplasia and cirrhosis (Azar et al., 2002). Knockdown of homologs of *JAG1* and other BA risk genes (*GPC1*, *ARF6*, *ADD3*, and *MAN1A2*) (Cui et al., 2013; Ningappa et al., 2015; Tang et al., 2016; So et al., 2020) showed the paucity of intrahepatic bile ducts. These lines of evidence supported that the alteration of intrahepatic bile ducts was a primary lesion in BA infants.

*JAG1* encodes the jagged 1 protein, which is the ligand for the receptor notch 1 involved in signaling processes. Notch signaling is an evolutionarily conserved intercellular signaling pathway which regulates the fate and differentiation of cells (Fortini, 2009), and the development and repair of bile ducts (Sparks et al., 2010). JAG1-mediated Notch signaling pathway is critical for postnatal bile duct growth and bile duct branching (Loomes et al., 2007; Roos et al., 2022). The target gene *Hes1* of Notch signaling contributes to the maturation and cytoarchitectonic organization of biliary epithelial cells (Zhang et al., 2018). Therefore, it is reasonable that JAG1 leads to or aggravates the development of BA by affecting the primitive development of hepatobiliary ducts. JAG1 is also involved in the inflammatory processes. JAG1-mediated Notch signaling regulates IL-8 production and inflammatory injury by HSF1/Snail and NLRP3 inflammasome activation in the liver (Kohsaka et al., 2002; Jin et al., 2020). NLRP3 regulates the bile duct obstruction in experimental BA (Yang et al., 2018). The interaction between Notch and NF- κB plays an important role in immune system development and the immune system response to inflammation and viral infection (Wang et al., 2001). *Jag1* is expressed in the biliary epithelium (Loomes et al., 2002). Thus, it could be speculated that JAG1 regulates inflammatory damage in the hepatic bile ducts, thereby causing or exacerbating BA. Since the primary disorder of bile duct development (Zani et al., 2015) and the continued activation of immune inflammatory processes (Bezerra et al., 2002; Wang et al., 2020) were the main features of BA, *JAG1* might contribute to BA by regulating the development of bile duct and inflammation in liver and bile duct.

ALGS and BA have some overlapping clinical features. The pathologic characteristics of ALGS differed from those of BA cases with intrahepatic biliary hyperplasia and obstructed extrahepatic bile ducts (Kohsaka et al., 2002; Han et al., 2022). Germline mutations of JAG*1* cause ALGS (Oda et al., 1997; Mitchell et al., 2018); however, mutations of *JAG1* were also found in BA and common SNPs were associated with sporadic BA in the current result. There are several explanations for these results. First, the decreased expression of *JAG1* caused by associated SNPs or BA mutations results in intrahepatic duct paucity. The paucity of bile ducts was likely to lead to cholestasis, which stimulated the proliferation of intrahepatic bile ducts (Tharehalli et al., 2018). The proliferation of intrahepatic bile ductular epithelium was increased in livers of BA patients (Kinugasa et al., 1999). The increased number of intrahepatic bile duct epithelial cells was found to be a poor prognosis factor for BA, and the density of proliferating cells was higher in patients with poor bile drainage than those of good bile drainage (Kinugasa et al., 1999). Therefore, it could be reasoned that common variation or mutations might work as an aggravation factor of BA, but as a casual factor of AGS. Second, ALGS mutations and BA mutations domains distributed in different functional domains of JAG1 (Kohsaka et al., 2002). The mutation with less harmful effects might impair the expression of *JAG1* and contribute to the development of BA. However, the deleterious *jag1* mutations could lead to failure of liver regeneration and prevent bile duct proliferation (Zhao et al., 2022), which causes ALGS.

## 5 Conclusion

In summary, our study found that common variation of *JAG1* was associated with susceptibility of BA in the Chinese Han population. Knockdown of *jag1* led to defective bile duct formation and dysfunction of bile ducts in zebrafish model, which indicated that *JAG1* implicated in the etiology of BA. The current findings revealed new etiologic factors for BA development.

## Data Availability

The original contributions presented in the study are included in the article/Supplementary Material, further inquiries can be directed to the corresponding authors.

## References

[B1] Asai A. Miethke A. Bezerra J. A. (2015). Pathogenesis of biliary atresia: Defining biology to understand clinical phenotypes. Nat. Rev. Gastroenterol. Hepatol. 12 (6), 342–352. 10.1038/nrgastro.2015.74 26008129 PMC4877133

[B2] Azar G. Beneck D. Lane B. Markowitz J. Daum F. Kahn E. (2002). Atypical morphologic presentation of biliary atresia and value of serial liver biopsies. J. Pediatr. Gastroenterol. Nutr. 34 (2), 212–215. 10.1097/00005176-200202000-00020 11840042

[B3] Bai M .R. Niu W. B. Zhou Y. Gong Y. M. Lu Y. J. Yu X. X. (2020). Association of common variation in ADD3 and GPC1 with biliary atresia susceptibility. Aging (Albany NY) 12 (8), 7163–7182. 10.18632/aging.103067 32315284 PMC7202506

[B4] Berauer J. P. Mezina A. I. Okou D. T. Sabo A. Muzny D. M. Gibbs R. A. (2019). Identification of polycystic kidney disease 1 like 1 gene variants in children with biliary atresia splenic malformation syndrome. Hepatology 70 (3), 899–910. 10.1002/hep.30515 30664273 PMC6642859

[B5] Bezerra J. A. Tiao G. Ryckman F. C. Alonso M. Sabla G. E. Shneider B. (2002). Genetic induction of proinflammatory immunity in children with biliary atresia. Lancet 360 (9346), 1653–1659. 10.1016/S0140-6736(02)11603-5 12457789

[B6] Bezerra J. A. Wells R. G. Mack C. L. Karpen S. J. Hoofnagle J. H. Doo E. (2018). Biliary atresia: Clinical and research challenges for the twenty-first century. Hepatology 68 (3), 1163–1173. 10.1002/hep.29905 29604222 PMC6167205

[B7] Chen Y. Gilbert M. A. Grochowski C. M. McEldrew D. Llewellyn J. Waisbourd-Zinman O. (2018). A genome-wide association study identifies a susceptibility locus for biliary atresia on 2p16.1 within the gene EFEMP1. PLoS Genet. 14 (8), e1007532. 10.1371/journal.pgen.1007532 30102696 PMC6107291

[B8] Cheng G. Tang C. S. Wong E. H. Cheng W. W. C. So M. T. Miao X. (2013). Common genetic variants regulating ADD3 gene expression alter biliary atresia risk. J. Hepatol. 59 (6), 1285–1291. 10.1016/j.jhep.2013.07.021 23872602

[B9] Cheng G. Chung P. H. Chan E. K. So M. T. Sham P. C. Cherny S. S. (2017). Patient complexity and genotype-phenotype correlations in biliary atresia: A cross-sectional analysis. BMC Med. Genomics 10 (1), 22. 10.1186/s12920-017-0259-0 28416017 PMC5392958

[B10] Chiu C. Y. Chen P. H. Chan C. F. Chang M. H. Wu T. C. Taiwan Infant Stool Color Card Study Group (2013). Biliary atresia in preterm infants in taiwan: A nationwide survey. J. Pediatr. 163 (1), 100–103.e1. 10.1016/j.jpeds.2012.12.085 23414661

[B11] Chu A. S. Russo P. A. Wells R. G. (2012). Cholangiocyte cilia are abnormal in syndromic and non-syndromic biliary atresia. Mod. Pathol. 25 (5), 751–757. 10.1038/modpathol.2011.212 22301700 PMC3341539

[B12] Cui S. Leyva-Vega M. Tsai E. A. EauClaire S. F. Glessner J. T. Hakonarson H. (2013). Evidence from human and zebrafish that GPC1 is a biliary atresia susceptibility gene. Gastroenterology 144 (5), 1107–1115. 10.1053/j.gastro.2013.01.022 23336978 PMC3736559

[B13] Feldman A. G. Sokol R. J. (2019). Neonatal cholestasis: Emerging molecular diagnostics and potential novel therapeutics. Nat. Rev. Gastroenterol. Hepatol. 16 (6), 346–360. 10.1038/s41575-019-0132-z 30903105

[B14] Fortini M. E. (2009). Notch signaling: The core pathway and its posttranslational regulation. Dev. Cell. 16 (5), 633–647. 10.1016/j.devcel.2009.03.010 19460341

[B15] Garcia-Barceló M. M. Yeung M. Y. Miao X. P. Tang C. S. M. Cheng G. Chen G. (2010). Genome-wide association study identifies a susceptibility locus for biliary atresia on 10q24.2. Hum. Mol. Genet. 19 (14), 2917–2925. 10.1093/hmg/ddq196 20460270 PMC2893814

[B16] Han Y. Zhu K. Wu H. Chen B. Hu S. Lai D. (2022). Case Report: Novel JAG1 gene mutations in two infants with alagille syndrome characterized by cholestasis. Front. Pediatr. 10, 1017647. 10.3389/fped.2022.1017647 36340723 PMC9631024

[B17] Hartley J. L. Davenport M. Kelly D. A. (2009). Biliary atresia. Lancet (London, Engl. 374 (9702), 1704–1713. 10.1016/S0140-6736(09)60946-6 19914515

[B18] Jin Y. Li C. Xu D. Zhu J. Wei S. Zhong A. (2020). Jagged1-mediated myeloid Notch1 signaling activates HSF1/Snail and controls NLRP3 inflammasome activation in liver inflammatory injury. Cell. Mol. Immunol. 17 (12), 1245–1256. 10.1038/s41423-019-0318-x 31673056 PMC7784844

[B19] Kinugasa Y. Nakashima Y. Matsuo S. Shono K. Suita S. Sueishi K. (1999). Bile ductular proliferation as a prognostic factor in biliary atresia: An immunohistochemical assessment. J. Pediatr. Surg. 34 (11), 1715–1720. 10.1016/s0022-3468(99)90652-8 10591578

[B20] Kohsaka T. Yuan Z. R. Guo S. X. Tagawa M. Nakamura A. Nakano M. (2002). The significance of human jagged 1 mutations detected in severe cases of extrahepatic biliary atresia. Hepatology 36 (4), 904–912. 10.1053/jhep.2002.35820 12297837

[B21] Lakshminarayanan B. Davenport M. (2016). Biliary atresia: A comprehensive review. J. Autoimmun. 73, 1–9. 10.1016/j.jaut.2016.06.005 27346637

[B22] Lam W. Y. Tang C. S. So M. T. Yue H. Hsu J. S. Chung P. H. Y. (2021). Identification of a wide spectrum of ciliary gene mutations in nonsyndromic biliary atresia patients implicates ciliary dysfunction as a novel disease mechanism. EBioMedicine 71, 103530. 10.1016/j.ebiom.2021.103530 34455394 PMC8403738

[B23] Leyva-Vega M. Gerfen J. Thiel B. D. Jurkiewicz D. Rand E. B. Pawlowska J. (2010). Genomic alterations in biliary atresia suggest region of potential disease susceptibility in 2q37.3. Am. J. Med. Genet. Part A 152a (4), 886–895. 10.1002/ajmg.a.33332 20358598 PMC2914625

[B24] Li S. X. Zhang Y. Sun M. Shi B. Xu Z. Y. Huang Y. (2008). Ultrasonic diagnosis of biliary atresia: A retrospective analysis of 20 patients. World J. Gastroenterol. 14 (22), 3579–3582. 10.3748/wjg.14.3579 18567090 PMC2716624

[B25] Loomes K. M. Taichman D. B. Glover C. L. Williams P. T. Markowitz J. E. Piccoli D. A. (2002). Characterization of Notch receptor expression in the developing mammalian heart and liver. Am. J. Med. Genet. 112 (2), 181–189. 10.1002/ajmg.10592 12244553

[B26] Loomes K. M. Russo P. Ryan M. Nelson A. Underkoffler L. Glover C. (2007). Bile duct proliferation in liver-specific Jag1 conditional knockout mice: Effects of gene dosage. Hepatol. Baltim. Md) 45 (2), 323–330. 10.1002/hep.21460 17366661

[B27] Louis A. A. Van Eyken P. Haber B. A. Hicks C. Weinmaster G. Taub R. (1999). Hepatic jagged1 expression studies. Hepatol. Baltim. Md) 30 (5), 1269–1275. 10.1002/hep.510300512 10534349

[B28] Lu Y. J. Yu W. W. Cui M. M. Yu X. X. Song H. L. Bai M. R. (2021). Association analysis of variants of DSCAM and BACE2 with hirschsprung disease susceptibility in han Chinese and functional evaluation in zebrafish. Front. Cell. Dev. Biol. 9, 641152. 10.3389/fcell.2021.641152 34136475 PMC8201997

[B29] Malik A. Thanekar U. Mourya R. Shivakumar P. (2020). Recent developments in etiology and disease modeling of biliary atresia: A narrative review. Dig. Med. Res. 3, 59. 10.21037/dmr-20-97 33615212 PMC7891552

[B30] Matthews R. P. Arias I. M. Alter H. J. Boyer J. L. Cohen D. E. Fausto N. (2009). “Zebra as a model system for the study of liver development and disease,” in The liver: Biology and pathobiology. 5th ed274 (Hoboken, NJ: Wiley), 245–259.

[B31] McCurley A. T. Callard G. V. (2008). Characterization of housekeeping genes in zebrafish: Male-female differences and effects of tissue type, developmental stage and chemical treatment. BMC Mol. Biol. 9, 102. 10.1186/1471-2199-9-102 19014500 PMC2588455

[B32] Mitchell E. Gilbert M. Loomes K. M. (2018). Alagille syndrome. Clin. liver Dis. 22 (4), 625–641. 10.1016/j.cld.2018.06.001 30266153

[B33] Ningappa M. So J. Glessner J. Ashokkumar C. Ranganathan S. Min J. (2015). The role of ARF6 in biliary atresia. PLoS One 10 (9), e0138381. 10.1371/journal.pone.0138381 26379158 PMC4574480

[B34] Oda T. Elkahloun A. G. Pike B. L. Okajima K. Krantz I. D. Genin A. (1997). Mutations in the human Jagged1 gene are responsible for Alagille syndrome. Nat. Genet. 16 (3), 235–242. 10.1038/ng0797-235 9207787

[B35] Ong C. T. Corces V. G. (2011). Enhancer function: New insights into the regulation of tissue-specific gene expression. Nat. Rev. Genet. 12 (4), 283–293. 10.1038/nrg2957 21358745 PMC3175006

[B36] Roos F. J. M. van Tienderen G. S. Wu H. Bordeu I. Vinke D. Albarinos L. M. (2022). Human branching cholangiocyte organoids recapitulate functional bile duct formation. Cell. stem Cell. 29 (5), 776–794.e13. 10.1016/j.stem.2022.04.011 35523140

[B37] Sangkhathat S. Laochareonsuk W. Maneechay W. Kayasut K. Chiengkriwate P. (2018). Variants associated with infantile cholestatic syndromes detected in extrahepatic biliary atresia by whole exome studies: A 20-case series from Thailand. J. Pediatr. Genet. 7 (2), 67–73. 10.1055/s-0038-1632395 29707407 PMC5916803

[B38] Schoenfelder S. Fraser P. (2019). Long-range enhancer-promoter contacts in gene expression control. Nat. Rev. Genet. 20 (8), 437–455. 10.1038/s41576-019-0128-0 31086298

[B39] So J. Khaliq M. Evason K. Ninov N. Martin B. L. Stainier D. Y. R. (2018). Wnt/β-catenin signaling controls intrahepatic biliary network formation in zebrafish by regulating notch activity. Hepatology 67 (6), 2352–2366. 10.1002/hep.29752 29266316 PMC5991997

[B40] So J. Ningappa M. Glessner J. Min J. Ashokkumar C. Ranganathan S. (2020). Biliary-atresia-associated mannosidase-1-alpha-2 gene regulates biliary and ciliary morphogenesis and laterality. Front. Physiol. 11, 538701. 10.3389/fphys.2020.538701 33192543 PMC7662016

[B41] Sokol R. J. Shepherd R. W. Superina R. Bezerra J. A. Robuck P. Hoofnagle J. H. (2007). Screening and outcomes in biliary atresia: Summary of a national institutes of health workshop. Hepatol. Baltim. Md) 46 (2), 566–581. 10.1002/hep.21790 PMC3888317 17661405

[B42] Sparks E. E. Huppert K. A. Brown M. A. Washington M. K. (2010). Notch signaling regulates formation of the three-dimensional architecture of intrahepatic bile ducts in mice. Hepatol. Baltim. Md) 51 (4), 1391–1400. 10.1002/hep.23431 PMC2995854 20069650

[B43] Tang V. Cofer Z. C. Cui S. Sapp V. Loomes K. M. Matthews R. P. (2016). Loss of a candidate biliary atresia susceptibility gene, add3a, causes biliary developmental defects in zebrafish. J. Pediatr. Gastroenterol. Nutr. 63 (5), 524–530. 10.1097/MPG.0000000000001375 27526058 PMC5074882

[B44] Tharehalli U. Svinarenko M. Kraus J. M. Kühlwein S. D. Szekely R. Kiesle U. (2018). YAP activation drives liver regeneration after cholestatic damage induced by rbpj deletion. Int. J. Mol. Sci. 19 (12), 3801. 10.3390/ijms19123801 30501048 PMC6321044

[B45] Verkade H. J. Bezerra J. A. Davenport M. Schreiber R. A. Mieli-Vergani G. Hulscher J. B. (2016). Biliary atresia and other cholestatic childhood diseases: Advances and future challenges. J. Hepatol. 65 (3), 631–642. 10.1016/j.jhep.2016.04.032 27164551

[B46] Wada H. Muraji T. Yokoi A. Okamoto T. Sato S. Takamizawa S. (2007). Insignificant seasonal and geographical variation in incidence of biliary atresia in Japan: A regional survey of over 20 years. J. Pediatr. Surg. 42 (12), 2090–2092. 10.1016/j.jpedsurg.2007.08.035 18082714

[B47] Wang J. Shelly L. Miele L. Boykins R. Norcross M. A. Guan E. (2001). Human Notch-1 inhibits NF-kappa B activity in the nucleus through a direct interaction involving a novel domain. J. Immunol. 167 (1), 289–295. 10.4049/jimmunol.167.1.289 11418662

[B48] Wang J. Xu Y. Chen Z. Liang J. Lin Z. Liang H. (2020). Liver immune profiling reveals pathogenesis and therapeutics for biliary atresia. Cell. 183 (7), 1867–1883.e26. 10.1016/j.cell.2020.10.048 33248023

[B49] Yang L. Mizuochi T. Shivakumar P. Mourya R. Luo Z. Gutta S. (2018). Regulation of epithelial injury and bile duct obstruction by NLRP3, IL-1R1 in experimental biliary atresia. J. hepatology 69 (5), 1136–1144. 10.1016/j.jhep.2018.05.038 PMC6314850 29886157

[B50] Zani A. Quaglia A. Hadzić N. Zuckerman M. Davenport M. (2015). Cytomegalovirus-associated biliary atresia: An aetiological and prognostic subgroup. J. Pediatr. Surg. 50 (10), 1739–1745. 10.1016/j.jpedsurg.2015.03.001 25824438

[B51] Zhang R. Z. Zeng X. H. Lin Z. F. Fu M. Tong Y. L. Lui V. C. (2018). Downregulation of Hes1 expression in experimental biliary atresia and its effects on bile duct structure. World J. Gastroenterol. 24 (29), 3260–3272. 10.3748/wjg.v24.i29.3260 30090006 PMC6079292

[B52] Zhao C. Matalonga J. Lancman J. J. Liu L. Xiao C. Kumar S. (2022). Regenerative failure of intrahepatic biliary cells in Alagille syndrome rescued by elevated Jagged/Notch/Sox9 signaling. Proc. Natl. Acad. Sci. U. S. A. 119 (50), e2201097119. 10.1073/pnas.2201097119 36469766 PMC9897440

