# Supplementary material for: Association analysis and functional follow-up identified common variants of JAG1 accounting for risk to biliary atresia
Source: Front Genet. 2023 May 15;14:1186882. doi: 10.3389/fgene.2023.1186882 (PMC10225652; doi:10.3389/fgene.2023.1186882)
Supplement: Supplementary file 1 [file Table1.DOCX]

Supplementary Material

# Supplementary Tables

**Supplementary Table 1. Allelic association test of 31 tag SNPs in 333 unrelated sporadic BA patients and 1,665 controls.**

| SNP | BP | Functional annotation | Reference allele | | Alternative allele | RAF | | Cases *vs* controls | | |
| --- | --- | --- | --- | --- | --- | --- | --- | --- | --- | --- |
|  |  |  |  |  |  | Cases | Controls |  | *P* | OR 95%CI |
| rs1232598 | 10600436 | 5' upstream | C | A | | 0.378 | 0.375 |  | 0.85 | 1.02 (0.86-1.21) |
| rs7270295 | 10602404 | 5' upstream | A | G | | 0.191 | 0.190 |  | 0.96 | 1.01 (0.81-1.24) |
| rs6133979 | 10605004 | 5' upstream | G | A | | 0.640 | 0.639 |  | 0.96 | 1.00 (0.84-1.19) |
| rs1232607 | 10609594 | 5' upstream | C | T | | 0.889 | 0.859 |  | 0.04 | 1.32 (1.02-1.71) |
| rs16991867 | 10609640 | 5' upstream | C | T | | 0.812 | 0.785 |  | 0.11 | 1.19 (0.96-1.47) |
| rs1232605 | 10611063 | 5' upstream | A | G | | 0.643 | 0.616 |  | 0.19 | 1.12 (0.94-1.34) |
| rs1232603 | 10612963 | 5' upstream | T | C | | 0.143 | 0.131 |  | 0.43 | 1.10 (0.87-1.40) |
| rs6074162 | 10613270 | 5' upstream | A | G | | 0.981 | 0.978 |  | 0.70 | 1.13 (0.62-2.04) |
| rs79905819 | 10616168 | 5' upstream | A | C | | 0.042 | 0.036 |  | 0.48 | 1.16 (0.77-1.77) |
| rs7828 | 10619014 | intron | A | C | | 0.857 | 0.852 |  | 0.74 | 1.04 (0.82-1.32) |
| rs35761929 | 10622501 | nsSNP | C | G | | 0.044 | 0.038 |  | 0.51 | 1.15 (0.76-1.73) |
| rs3817996 | 10624046 | intron | G | A | | 0.863 | 0.856 |  | 0.60 | 1.07 (0.84-1.36) |
| rs6077861 | 10624926 | intron | A | T | | 0.923 | 0.8717 |  | 1.74×10^-4^ | 1.78 (1.31-2.40) |
| rs34325313 | 10629129 | intron | C | A | | 0.245 | 0.224 |  | 0.24 | 1.12 (0.92-1.36) |
| rs35793014 | 10632577 | intron | G | A | | 0.993 | 0.988 |  | 0.31 | 1.61 (0.63-4.09) |
| rs6040055 | 10633313 | intron | A | G | | 0.500 | 0.494 |  | 0.78 | 1.02 (0.87-1.21) |
| rs1801138 | 10639222 | intron | G | A | | 0.809 | 0.792 |  | 0.32 | 1.11 (0.90-1.37) |
| rs2273061 | 10639543 | intron | G | A | | 0.350 | 0.342 |  | 0.69 | 1.04 (0.87-1.23) |
| rs3790160 | 10639988 | intron | A | G | | 0.300 | 0.297 |  | 0.86 | 1.02 (0.85-1.22) |
| rs17457340 | 10640042 | intron | T | C | | 0.989 | 0.985 |  | 0.35 | 1.46 (0.66-3.23) |
| rs6040060 | 10640201 | intron | G | A | | 0.856 | 0.849 |  | 0.65 | 1.06 (0.83-1.34) |
| rs3748478 | 10641575 | intron | T | C | | 0.286 | 0.224 |  | 5.77×10^-4^ | 1.39 (1.15-1.67) |
| rs3748479 | 10641633 | intron | A | C | | 0.463 | 0.456 |  | 0.75 | 1.03 (0.87-1.21) |
| rs6133986 | 10645773 | intron | A | G | | 0.030 | 0.028 |  | 0.73 | 1.09 (0.67-1.78) |
| rs3790163 | 10647951 | intron | G | A | | 0.884 | 0.860 |  | 0.09 | 1.25 (0.97-1.61) |
| rs73604316 | 10648706 | intron | A | G | | 0.245 | 0.226 |  | 0.28 | 1.11 (0.92-1.35) |
| rs910119 | 10656059 | 3' downstream | A | T | | 0.987 | 0.970 |  | 0.02 | 2.24 (1.13-4.45) |
| rs74711724 | 10658248 | 3' downstream | G | A | | 0.982 | 0.981 |  | 0.84 | 1.07 (0.57-1.99) |
| rs10485742 | 10665206 | 3' downstream | C | A | | 0.272 | 0.240 |  | 0.08 | 1.18 (0.98-1.43) |
| rs2423510 | 10670079 | 3' downstream | T | A | | 0.478 | 0.466 |  | 0.59 | 1.05 (0.89-1.24) |
| rs6074170 | 10671078 | 3' downstream | G | A | | 0.887 | 0.881 |  | 0.65 | 1.06 (0.82-1.38) |

SNP: single nucleotide polymorphism; nsSNP: nonsynonymous single nucleotide polymorphism; BP: base pair; RAF: risk allele frequency; OR: odds ratio; CI: confidence interval.

**Supplementary Table 2. Genotypic association test of 31 tag SNPs in 333 unrelated sporadic BA patients and 1,665 controls.**

|  | Allele | |  | A1A1/A1A2/A2A2 | |  | Cases vs controls |
| --- | --- | --- | --- | --- | --- | --- | --- |
| SNP | A1 | A2 |  | Cases | Controls |  | *P* |
| rs1232598 | C | A |  | 50/152/131 | 247/751/664 |  | 0.98 |
| rs7270295 | A | G |  | 9/109/215 | 71/490/1104 |  | 0.24 |
| rs6133979 | G | A |  | 133/159/40 | 683/761/220 |  | 0.73 |
| rs1232607 | C | T |  | 266/60/7 | 1231/397/37 |  | 0.07 |
| rs16991867 | C | T |  | 224/93/16 | 1033/547/85 |  | 0.19 |
| rs1232605 | A | G |  | 135/158/40 | 625/799/240 |  | 0.40 |
| rs1232603 | T | C |  | 11/73/249 | 29/379/1256 |  | 0.18 |
| rs6074162 | A | G |  | 321/11/1 | 1593/69/2 |  | 0.58 |
| rs79905819 | A | C |  | 2/24/307 | 2/117/1546 |  | 0.20 |
| rs7828 | A | C |  | 241/87/4 | 1208/416/38 |  | 0.43 |
| rs35761929 | C | G |  | 2/25/306 | 2/123/1540 |  | 0.20 |
| rs3817996 | G | A |  | 245/85/3 | 1222/405/38 |  | 0.25 |
| rs6077861 | A | T |  | 286/43/4 | 1263/375/26 |  | 3.22×10^-4^ |
| rs34325313 | C | A |  | 19/125/189 | 81/584/1000 |  | 0.50 |
| rs35793014 | G | A |  | 328/5/0 | 1625/40/0 |  | NA |
| rs6040055 | A | G |  | 73/186/73 | 406/833/426 |  | 0.13 |
| rs1801138 | G | A |  | 218/103/12 | 1049/539/76 |  | 0.60 |
| rs2273061 | G | A |  | 31/171/131 | 213/712/740 |  | 0.01 |
| rs3790160 | A | G |  | 25/150/158 | 164/661/840 |  | 0.13 |
| rs17457340 | T | C |  | 324/7/0 | 1609/51/0 |  | NA |
| rs6040060 | G | A |  | 242/86/5 | 1203/421/41 |  | 0.56 |
| rs3748478 | T | C |  | 19/152/161 | 73/600/991 |  | 9.65×10^-4^ |
| rs3748479 | A | C |  | 64/180/89 | 366/786/513 |  | 0.07 |
| rs6133986 | A | G |  | 0/20/313 | 0/92/1572 |  | NA |
| rs3790163 | G | A |  | 259/71/3 | 1229/405/31 |  | 0.21 |
| rs73604316 | A | G |  | 16/131/186 | 94/563/1008 |  | 0.15 |
| rs910119 | A | T |  | 324/9/0 | 1567/97/1 |  | NA |
| rs74711724 | G | A |  | 321/12/0 | 1601/64/0 |  | NA |
| rs10485742 | C | A |  | 26/129/178 | 101/598/966 |  | 0.23 |
| rs2423510 | T | A |  | 80/158/95 | 363/826/476 |  | 0.64 |
| rs6074170 | G | A |  | 262/67/4 | 1298/338/29 |  | 0.77 |

SNP: single nucleotide polymorphism.

**Supplementary Table 3. Functional annotation of SNPs in *JAG1* associated with BA using data from Haploreg v4.1.**

| **SNP** | **Position** | **Promoter**  **histone marks ^b^** | **Enhancer**  **histone**  **marks ^c^** | **DNAse ^d^** | **Motifs Changed ^e^** | **Selected eQTL Hits ^f^** |
| --- | --- | --- | --- | --- | --- | --- |
| rs6077861 | 10624926 |  | 8 tissues | SKIN, BLD | Cphx, Foxp1,  RREB-1 | 1 hit |
| rs3748478 | 10641575 | MUS | 18 tissues | 16 tissues | PPAR, VDR |  |

SNP: single nucleotide polymorphism; eQTL: expression quantitative trait loci; The chromosome position (bp) is based on GRCH37;

MUS: muscle satellite cultured cells; SKIN: foreskin fibroblast primary cells; BLD: peripheral blood; Cphx: cytoplasmic polyadenylated homeobox 1; Foxp1: forkhead box p1; RREB-1: ras responsive element binding protein 1; PPAR: peroxisome proliferator activated receptor; VDR: vitamin d receptor.

^b^ Evidence of local H3K4Me3 and H3K9Ac modification (cell lines/types: if >3, only the number is included).

^c^ Evidence of local H3K4Me1 and H3K27Ac modification (cell lines/types: if >3, only the number is included).

^d^ Evidence of chromatin hypersensitivity to DNase (cell lines/types: if >3, only the number is included).

^e^ Evidence of alteration in regulatory motif (if >3, only the number is included).

^f^ Evidence of eQTL studies (if >3, only the number is included).

**Supplementary Table 4. Primers used for q-PCR.**

| **Primer ID** | **Sequence** |
| --- | --- |
| *jag1a* forward primer-236bp | 5’- GAACCGGACCCAGATGTCAG-3’ |
| *jag1a* forward primer-236bp | 5’- GACGAAACACTGCTCATCGC-3’ |
| *jag1b* forward primer-179bp | 5’-GAACGCGCAGTGAAAAGTCA-3’ |
| *jag1b* forward primer-179bp | 5’-GAGCCTCTGCCATTGTCGAT-3’ |
| *18s* forward primer-62bp | 5’-TCGCTAGTTGGCATCGTTTATG-3’ |
| *18s* forward primer-62bp | 5’-CGGAGGTTCGAAGACGATCA-3’ |

RT-PCR, real time polymerase chain reaction; BP: base pair

**Supplementary Table 5. Primers used for RT-PCR.**

| **Primer ID** | **Sequence** |
| --- | --- |
| *jag1a* forward primer-226bp | 5’-CCTGCGACGAGCACTACTTT-3’ |
| *jag1a* forward primer-226bp | 5’-TATTCACCCTGCCACCCGTA-3’ |
| *jag1b* forward primer-195bp | 5’-ACTGGATCTACGCCCGTTCT-3’ |
| *jag1b* forward primer-195bp | 5’-CATGCCAGAGTGGTACGCTT-3’ |

Q-PCR: quantitative real time polymerase chain reaction; BP: base pair

**Supplementary Table 6. Primers used for *in situ* hybridization probe.**

| **Primer ID** | **Sequence** |
| --- | --- |
| *jag1a* forward primer-893bp | 5’- AACACCTGTGGCACACTTCA -3’ |
| *jag1a* forward primer-893bp | 5’- GAGATTAGCCTCAGACCGCC -3’ |
| *jag1b* forward primer-824bp | 5’- CAGGGCCTGACAAGTACCAG -3’ |
| *jag1b* forward primer-824bp | 5’- TTCCGGTGTACTGTTGGACG -3’ |

BP: base pair

**Supplementary Table 7. MOs sequences.**

| **Primer ID** | **Sequence** |
| --- | --- |
| standard control MO | 5’- CCTCTTACCTCAGTTACAATTTATA -3’ |
| *jag1a* splicing MO | 5’- AGACACCTATGAAGGGCACGACATC -3’ |
| *jag1a* translation blocking MO | 5’- TCGGTCTGAGAATCATCCCGGTTTG -3’ |
| *jag1b* splicing MO | 5’- AATCCTGCTACTCACTTTCACTGGC -3’ |
| *jag1b* translation blocking MO | 5’- AACTCCGTCGCAGAATCATGCCTTC -3’ |

MO: morpholino
